# Smartfeeding: A Dynamic Strategy to Increase Nutritional Efficiency in Critically Ill Patients—Positioning Document of the Metabolism and Nutrition Working Group and the Early Mobilization Working Group of the Catalan Society of Intensive and Critical Care Medicine (SOCMiC)

**DOI:** 10.3390/nu16081157

**Published:** 2024-04-13

**Authors:** Juan Carlos Yébenes, Maria Luisa Bordeje-Laguna, Juan Carlos Lopez-Delgado, Carol Lorencio-Cardenas, Itziar Martinez De Lagran Zurbano, Elisabeth Navas-Moya, Lluis Servia-Goixart

**Affiliations:** 1 Institut d’Assistència Sanitària (IAS)—Hospital Santa Caterina, 17007 Girona, Spain; 2 Hospital Universitari Germans Trias i Pujol, 08916 Badalona, Spain; luisabordeje@gmail.com; 3 Hospital Clinic, Medical ICU, Clinical Institute of Internal Medicine & Dermatology (ICMiD), 08036 Barcelona, Spain; juancarloslopezde@hotmail.com; 4 Hospital Universitari Dr. Josep Trueta, 17007 Girona, Spain; carol_lorencio@hotmail.com; 5 Hospital de Mataró, 08304 Mataró, Spain; 6 Hospital Universitari Vall d’Hebron, 08035 Barcelona, Spain; elisabeth.navas@vallhebron.cat; 7 Intensive Care Department, Hospital Universitari Arnau de Vilanova, LLeida, 25198 Lleida, Spain; lserviag@gmail.com

**Keywords:** intensive care units, critical illness, nutritional therapy, functional recovery

## Abstract

Adequate medical nutrition therapy for critically ill patients has an impact on their prognoses. However, it requires an individualized approach that takes into account the activity (phases of metabolic stress) and particularity of these patients. We propose a comprehensive strategy considering the patients’ nutritional status and the set of modifiable circumstances in these patients, in order to optimize/support nutritional efficiency: (1) A detailed anamnesis and an adequate initial nutritional assessment must be performed in order to implement medical nutrition therapy that is in line with the needs and characteristics of each patient. Furthermore, risks associated with refeeding syndrome, nutritrauma or gastrointestinal dysfunction must be considered and prevented. (2) A safe transition between nutrition therapy routes and between health care units will greatly contribute to recovery. The main objective is to preserve lean mass in critically ill patients, considering metabolic factors, adequate protein intake and muscle stimulation. (3) Continuous monitoring is required for the successful implementation of any health strategy. We lack precise tools for calculating nutritional efficiency in critically ill patients, therefore thorough monitoring of the process is essential. (4) The medical nutrition approach in critically ill patients is multidisciplinary and requires the participation of the entire team involved. A comprehensive strategy such as this can make a significant difference in the functional recovery of critically ill patients, but leaders must be identified to promote training, evaluation, analysis and feedback as essential components of its implementation, and to coordinate this process with the recognition of hospital management.

## 1. Introduction

The Smartfeeding project was conceived as a dynamic strategy to enhance the functional recovery of critically ill patients. It aims to foster collaboration among any and all healthcare workers who can have an influence on the efficiency of medical nutrition therapy received by critically ill patients during and after their stay in the intensive care unit (ICU). The strategy is led by the Metabolism and Nutrition Working Group of the Catalan Society of Intensive and Critical Care Medicine (GTMiN-SOCMiC), in collaboration with the Early Mobilization Working Group (GTMP-SOCMiC).

## 2. Methodology

GTMiN-SOCMiC invited 12 hospitals to attend a discussion and positioning meeting. Each hospital was to send three professionals involved in the nutritional management of critically ill patients, one of whom was to be an intensive care unit physician and GTMiN member, while the other two were to have other care profiles (physiotherapist, physical medicine and rehabilitation physician, nurse, speech therapist, pharmacist, endocrinologist, etc.) at the discretion of each center.

A face-to-face meeting was scheduled in two distinct sessions. In the first session, following a brief introduction, there were seven topics of discussion:

- Introduction: Why we provide nutrition therapy to critically ill patients.
- The changing nature of critically ill patients: How many critically ill patients are there in a critically ill patient?
- The quality of the nutritional process: Calculate, prescribe... and administer!
- Does more always mean better? Safety in prescribing medical nutrition therapy.
- Is it enough for the patient to just eat? Determinants of lean-mass accretion.
- Is it possible to monitor nutritional efficiency?
- Key elements transforming a care process.

Attendees were distributed into tables of eight participants who were necessarily from different hospitals and had different care profiles. Each discussion topic was introduced with a brief overview (10 min), and the objectives, strengths and weaknesses associated with each topic were proposed to the tables to be discussed. The tables discussed each topic internally for 30 min. After the discussion, each table agreed on a series of recommendations in 15 min that were sent in writing to a central table where all recommendations from all tables were integrated.

In the second session, the groups were dissolved and the degree of consensus that the recommendations commanded was assessed through an interactive voting system (Kahoot! [1]). The following degrees of consensus were defined [2]:

- Strong consensus: Agreement of >90% of participants.
- Consensus: Agreement of 75–90% of participants.
- Majority agreement: Agreement of 50–75% of participants.
- No agreement: Agreement of <50% of participants.

After each vote, when the degree of consensus was less than strong, it was discussed and the conclusion was modified based on new assessments and input.

The conclusions were sent to the participants for validation prior to the drafting of the positioning document. The 40 recommendations agreed by the meeting participants can be found in Table 1. 

## 3. Conference Sections

**a. Why we provide nutrition therapy to critically ill patients**

Recovery from functional impairment in critically ill patients suffering from multi-organ failure is a challenge that requires the intervention of multiple participants, including critically ill patients themselves. This already complex scenario is aggravated by the demographic trend of an aging population, which is expected to significantly increase the number of elderly patients requiring ICU care [3].

A specific strategy for action is defined for each patient, with his/her own baseline status, weight, height, age and injury determining his/her critical status. If we were to describe it as a mathematical function, we could identify three components of a polynomial: nutritional risk associated with the patient’s baseline status, injury and nutritional therapy.

F(x) = (1 − Nutritional Risk) − (Injury) + (Nutritional Therapy)

In these circumstances, there would be factors that would fortify each of the above factors (muscle relaxants, corticosteroid treatment, new infections, extracorporeal techniques, loss of enteral route, early mobilization, etc.).

F(x) = (1 − Nutritional Risk) − (Injury × Sarcopenia Risk Factors) + (Nutritional Therapy × Anabolic Factors)

If we add the individual susceptibility of each patient (an individual constant, Ki) that would explain the different responses of patients to the same stimuli (or the same injury), the polynomial would read as follows:

F(x) = {(1 − Nutritional Risk) − (Injury × Sarcopenia Risk Factors) + (Nutritional Treatment × Anabolic Factors)} × Ki

But once this polynomial is constructed, the underlying question would be, What is the purpose of that function? What does F(x) mean in critically ill patients? What is that polynomial equivalent to? Perhaps to survival, to the length of their stay in the ICU, to the duration of weaning, to muscle strength, etc.?

Nutritional efficiency in critically ill patients could be defined as the recovery of the body’s functional lean mass, both muscular and visceral, based on nutritional therapy and contributing modifiable circumstances. 

In a patient with low nutritional risk and minimal injury, the margin is high; therefore, nutritional therapy and supportive anabolic measures, such as increased initial protein contribution, may not be significant [4,5,6]. In fact, patients will tolerate fasting and recover intake capacity, and it is highly likely that they will be able to return to their previous functional status [7,8], but this may not be the outlook for the patients we are discussing here.

Moreover, critically ill patients with nutritional risk who suffer an injury experience a systemic response that will develop over time, the impact of which will vary with different treatments (hemodiafiltration, mechanical ventilation, ECMO, surgical revisions, etc.), and it may or may not be possible to preserve the enteral route in these patients, and therefore intestinal function in all its complexity, and this will always determine nutritional objectives. Therefore, in terms of medical nutrition management, treatment for each patient must be part of a strategy and objectives in line with the baseline status and the development of the injury.

A strategy designed to bolster the efficiency of medical nutrition therapy in critically ill patients in general should capture the particularities of each center. The circuits of care and prescribing responsibilities are different in each unit or hospital. Ignoring the particularities, deficits or strengths of the different care models reduces a strategy’s effectiveness. However, considering the objective and how to reach it does indeed seem unique and particular to us. That is why we are inviting everyone who forms part of one or another of the modifiable variables in the equation to share the real difficulties they have in their routine clinical practice, to find common solutions and to emphasize those factors that enhance the patient’s functional recovery from a feasible and operational point of view.

We have called this strategy SMARTFEEDING. We are looking for a dynamic, progressive and continuous nutritional design that seeks to understand the unique and changing nature of critically ill patients, uses non-nutritional resources and is maintained over time when a patient moves from a critical to a fragile condition.

**b. The changing nature of critically ill patients: How many critically ill patients are there in a critically ill patient?**

**Figure 1:**
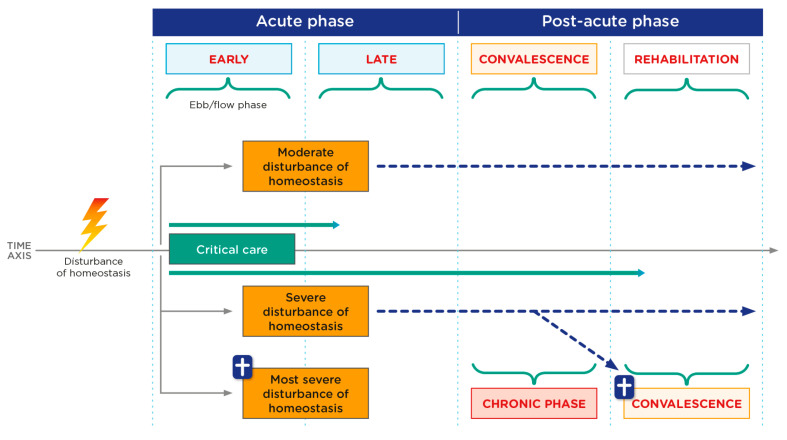
Critical patient phases. Phases of critical illness according to severity of disturbance of homeostasis. Adapted from Elke G, et al. [9].

Medical nutrition therapy should be a dynamic process and one that is adjusted to the different phases of metabolic stress (see Table 2). Critically ill patients are subjected to different types of injury and experience a systemic inflammatory response that results in metabolic stress. This response is acutely divided into an early phase (ebb) with shock (1–2 days) and a later phase (flow) of less determined duration and which is very dependent on the management of the initial phase (see Figure 1). 

Through a “second hit” (a new disturbance of homeostasis), a step backwards from the post-acute to the acute phase is possible at any time. The individual course of critical illness must be considered in each patient at all times with regard to the inflammatory and metabolic changes or changes in organ dysfunction, respectively.

Paradoxically, the nutritional goal in the acute phase is to avoid overfeeding since, in response to injury, a large number of endogenous substrates are produced from the patient’s [10] reserves [8,9,10], whereas in the stable phase, exogenous supply needs to be increased in order to avoid underfeeding. This later phase is characterized by a hypercatabolic state that typically lasts for the first seven days and, if the injury is controlled, there follows a subsequent phase of reconstruction in which an anabolic phase occurs. 

It should be noted that some patients suffer a persistent inflammation, immunosuppression and catabolism syndrome (PICS) at this later stage [6,11]. This is a dynamic process that may be conditioned by new injuries (reinfections, complications, surgical revisions, etc.), thereby changing the injury phase the patient is in, including a well-known state in which protein synthesis is blocked in persistent inflammatory processes.

On the other hand, we have to take into account the negative effects that different life support techniques used to keep patients alive in their fight against injury can have on lean mass, including lean mass loss associated with muscle rest, which can be extrapolated to loss of intestinal function associated with digestive rest, as well as the depleting effect of extracorporeal techniques, oversedation or immobility. These procedures or scenarios may overlap with the different physiological phases the patient is in. 

**c. The quality of the nutritional process: Calculate, prescribe... and administer!**

The prescription of medical nutrition therapy must be a directed process in which a detailed medical history (prior weight loss, days without oral intake, physical activity, digestive system drugs, etc.) is required and an adequate baseline nutritional assessment must be recorded in the patient’s medical record. On the other hand, nutrition therapy is a dynamic and continuous process over time that requires a contribution of both macronutrients and micronutrients (trace elements and vitamins) [11,12], taking into account the non-nutritional calories (glucose serum, propofol, citrate) provided. In light of the significant risk that malnutrition and underfeeding pose for complications [11], the evaluation for medical nutrition therapy should ideally begin prior to ICU admission. This preemptive approach is supported, for example, by the ESPEN guidelines for Clinical Nutrition in Surgery, which recommends the administration of oral nutritional supplements (ONS) during the preoperative period to patients unable to meet their energy needs through regular diet, irrespective of their nutritional status [13]. It also encompasses the critical consideration of maintaining adequate hydration, ensuring patients do not face the compounded difficulties associated with dehydration during ICU care.

For the critically ill, the guidelines from the European Society for Parenteral and Enteral Nutrition (ESPEN) recommend the initiation of early enteral nutrition (within 48 h) if oral intake is not possible [11]. Of particular note are patients with high nutritional risk, especially those with previous malnutrition, in whom progression in caloric prescription should be slow and progressive to avoid refeeding syndrome by producing excessive intracellular substrate consumption (especially phosphorus) after an excessive supply of exogenous nutrients [11,14].

There is some consensus regarding the calculation of caloric and protein requirements. The gold standard for energy expenditure measurement is indirect calorimetry (IC) [15]. If this is not available, either the predictive formulas or the weight-based formulas, formulas that are available to everyone prescribing nutrition therapy in the ICU, will be used [16]. Calculations will be based on weight prior to ICU admission, taking into account that, in patients with obesity, calculations should be adjusted in accordance with the patient’s body mass index (BMI) [17]. Explicitly, according to the recommendations of the Metabolism and Nutrition Working Group (GTMyN) of SEMICYUC (Sociedad Española de Medicina Intensiva Crítica y Unidades Coronarias [Spanish Society of Critical Care Medicine and Coronary Units]), an adequate caloric intake in the first week of ICU admission would be to reach 70% of the IC measurement or between 15–20 kcal/kg/day [18]. After the initial phase, or in the subgroup of malnourished patients, it is recommended that 25 kcal/kg be given initially and increased to 30–35 kcal/kg in the anabolic phase [18]. In obese patients (BMI 30–50), it will be adjusted to 11–14 kcal/actual weight/day, and for BMI > 50, 22–25 kcal/kg adjusted weight per day [18]. Recommended lipid doses are between 0.7–1.3 g/kg/day or 25–40% of caloric intake. It is recommended that, in case of total parenteral nutrition (TPN), lipid emulsions enriched with fish oil should be used because they improve the omega-6/omega-3 ratio and have anti-inflammatory effects [18].

In the later anabolic phase or in patients with extracorporeal clearance techniques, the initial protein contribution will be 1.2–1.5 g/kg/day and will increase to 1.5–2 g/kg/day. Obese patients with BMI > 30 require high-dose proteins (1.8–2.5 g/kg ideal weight/day) [8]. While excessive caloric prescription has been associated with increased morbidity in critically ill patients, the administered protein doses actually seem to have an anabolizing effect. 

Administration of enteral nutrition is of choice in critically ill patients if oral feeding is not possible. Early enteral nutrition (EN) should be initiated within the first 48 h of progression, after the resuscitation phase and once a stable shock situation has been reached (mean systolic blood pressure ≥65 mmHg after adequate resuscitation, stabilized and/or decreasing lactate levels and doses of vasopressors, and improvement of systemic perfusion) [19]. If, after the acute phase (7–10 days), 70% of the enteral protein-caloric needs are not achieved, add-on parenteral nutrition (PN) should be considered [20]. If EN cannot be administered due to intestinal failure, especially in patients at nutritional risk, TPN should be administered in progressive doses [8,11]. Attempts should be made to maintain trophic enteral nutrition at 10–20 kcal/h for its potential positive effects on maintaining gastrointestinal structure and function.

In patients who tolerate oral intake, excluding those with dysphagia, there should be careful monitoring of the amount ingested, and patients who are candidates to receive supplements should be identified if the calculated requirements are not met. 

Despite the clarity with which clinical practice guidelines are expressed, one of the main limitations in nutrition therapy, especially enteral, is not the design of the treatment but rather the administration thereof. It is essential to try to implement strategies to maximize enteral input and tolerance. In the recently published multicenter Spanish ENPIC study, nutrient administration did not reach a mean intake of 16 kcal/kg/day and 0.81 g/kg/day protein, which is a figure observed even in clinical trials: a scenario that we all associate with excellence in care [21]. 

**d. Does more always mean better? Safety in prescribing medical nutrition therapy.**

Inadequate nutrition therapy has been directly associated with increased morbidity and mortality in critically ill patients [22]. In this regard, it is critical to take the following actions:

(1) **Perform an adequate nutritional assessment to optimally adjust nutritional requirements.** The clinical severity of critically ill patients per se causes them to be at nutritional risk regardless of their previous nutritional status, and therefore they can benefit from personalized and targeted medical nutrition therapy. A proper nutritional assessment aims to identify patients at risk of or in a state of malnutrition, who would especially benefit from optimal personalized nutrition therapy, as malnutrition is associated with increased morbidity, mortality and healthcare costs [23,24]. There is no validated tool for nutritional assessment in the ICU; however, a complete physical examination of the patient is advised and should be supported by a good medical history, determination of biochemical parameters such as albumin, pre-albumin and cholesterol, and an assessment of anthropometric data such as BMI or recent variations from usual weight [11,25].
(2) **Detect patients at risk of refeeding syndrome**. Refeeding syndrome is characterized by the acute onset of severe and potentially lethal metabolic and functional alterations resulting from the reintroduction of nutrients, especially carbohydrates, in patients with severe malnutrition. Although relatively uncommon, when it occurs, it causes increased morbidity and mortality in critically ill patients [26,27]. The presence of baseline factors such as cancer, prolonged hospital stays, alcoholism, anorexia nervosa, recent weight loss, poorly controlled diabetes or malabsorption syndromes may help to detect patients at risk of refeeding syndrome [26,27].
(3) **Initiate nutrition therapy at the appropriate time.** An accumulated caloric and protein deficit is associated with increased mortality in critically ill patients [22]. For this reason, early initiation of nutrition therapy is recommended in ICU patients (<48 h) as long as there are no contraindications [11,25]. This is a key aspect in the care of critically ill patients because, despite the different studies showing that enteral nutrition in critically ill patients with vasoactive drugs is safe [28], a non-negligible percentage of unstable patients experience splanchnic hypoperfusion and are at risk of mesenteric ischemia. For this reason, in order for nutrition therapy to be initiated, especially when enteral, the patient needs to be in a state of stabilized shock, defined as the presence of systolic blood pressure ≥65 mmHg after adequate resuscitation, improvement of systemic perfusion, decrease in tissue hypoperfusion markers such as lactate, and stability or decrease in doses of vasopressors.
(4) **Avoid or minimize nutritrauma.** Nutritrauma refers to any metabolic adverse effect that occurs as a result of inadequate medical nutrition therapy, such as refeeding syndrome, hypertriglyceridemia or hyperhydration [29]. Nutritrauma can appear at any time during medical nutrition therapy in ICUs, but it is more common in the earliest phases of nutrition initiation and in patients receiving parenteral nutrition. The occurrence of nutritrauma has been associated with increased morbidity and mortality in critically ill patients [26,27,30,31], making its prevention vital. To avoid or minimize nutritrauma, a number of preventive measures are recommended, such as: adjusting the protein-caloric contribution to the patient’s clinical condition and the degree of metabolic stress; detecting and taking into account extra-nutritional caloric intake; maintaining the enteral route wherever possible, given its benefits for the intestinal mucosa [32]; having an effective glycemic control protocol; and measuring the fluid balance or analyzing the liver, lipid and electrolyte levels frequently [33].
(5) **Avoid or minimize gastrointestinal dysfunction.** Gastrointestinal dysfunction is a common phenomenon in critically ill patients and it encompasses a number of mechanical alterations of the gastrointestinal tract such as increased gastric residue, vomiting, diarrhea, constipation or abdominal distension, which have been associated with increased morbidity and mortality [34,35]. There are multiple causes that favor gastrointestinal dysfunction in critically ill patients, such as hypoperfusion and ischemia of the digestive tract, the use of different drugs such as benzodiazepines, opioids or neuromuscular blockers, or intestinal dysbiosis, among others. One of the factors that we should not omit is alterations in the secretion of the different gastrointestinal hormones that are related to intestinal motility [34]. Therefore, it is essential to circumvent the predisposing factors discussed above and to monitor daily for any clinical signs indicating occurrence in order to initiate specific early treatment and to prevent its progression.
(6) **Ensure a safe transition when there are changes in the access route for nutrition therapy.** As a result of the constant clinical changes that critically ill patients present throughout their stay in the ICU, we often need to rethink nutrition therapy and make changes to the access route for nutrition. The time of transition from one route to another (enteral to parenteral, parenteral to enteral, or any of them to oral) is a sensitive time during which poor nutritional monitoring can lead to significant errors with important clinical consequences, such as over or underfeeding, hyperglycemia, hyperhydration, etc. For this reason, it is advisable to have a clear protocol with explicit guidelines on how to make a transition, as well as close monitoring of the onset of any of these complications so that they can be treated and their progression prevented. It is important to mention that one of the most common and serious complications associated with transitioning nutrition therapy to the oral route is aspiration secondary to dysphagia. Dysphagia is a common phenomenon in critically ill patients who have required mechanical ventilation and/or tracheostomy, and its presence has been associated with increased morbidity and mortality [36]. Early diagnosis of dysphagia is essential for planning targeted multidisciplinary treatment and implementing oral intake when it is safe. In this regard, screening for dysphagia is essential for all patients who have undergone orotracheal intubation for more than 48 h, patients who have a tracheostomy cannula who need to start an oral diet, or patients who have been decannulated before starting oral intake [36,37].
(7) **Ensure continuity of appropriate nutrition therapy on discharge from the ICU.** While it is true that specific and appropriate medical nutrition therapy in critically ill patients provides significant benefits to prognosis, when discharge from the ICU to other conventional inpatient services or rehabilitation centers is considered, the recovering patient requires more specific nutritional intake and in greater amounts than in previous phases in order to satisfy and consolidate the anabolism and muscle growth typical of this phase [38] However, the patient’s nutritional and functional recovery may be compromised upon discharge from the ICU if there is a lack of communication between different care teams and if the patient is not identified as being at risk. For this reason, the continuity and adequacy of nutrition therapy should be guaranteed during this period of development in the care of critically ill patients, and, to this end, teamwork is essential by means of a multidisciplinary group that understands and monitors the needs of this type of patient [38].

**e. Is it enough for the patient to just eat? Determinants of lean-mass accretion**

Preserving lean mass in critically ill patients is one of the main objectives of nutrition therapy. Patient immobilization itself involves a loss of lean mass. Its deficit may compromise immunological function, functional capacity and recovery time, and it can increase the mortality rate. The greater the loss of lean mass, the more difficult it is for the patient to recover. That is why the key point of the critically ill patient’s nutrometabolic treatment will be to slow down muscle destruction as much as possible so that food is transformed into lean mass for functional recovery [39]. Muscle proteins are in a constant state of renewal, and the balance between the rates of protein breakdown and synthesis determines whether there is a net gain (anabolism) or a net loss (catabolism, wasting).

Protein anabolism is going to depend on a number of factors, which are discussed in the following section.

### 3.1. Exit Phase of the Critical Situation

In the acute and stable phase of critical illness, protein catabolism will be increased supraphysiologically, and nutritional intervention will be aimed at preventing muscle proteolysis, if possible. The body implements mechanisms of protein synthesis that, at all times, will be insufficient to compensate for loss. Furthermore, in this phase, patients are inactive, which is partly due to the impact of disease severity, clinical instability, sedation, delirium and the concomitant treatments they are receiving to try to save their lives.

In the exit phase of critical illness, anabolism occurs, but it remains insufficient when it comes to compensating for all the protein degradation. Anabolic resistance occurs and is explained by three major factors: splanchnic sequestration of exogenous amino acids (AA), which decreases the AA available to muscles; insulin resistance, which limits the uptake of AA in muscles and makes muscle protein maintenance difficult; and attenuated responses to AA with anabolic properties such as leucine. In this anabolic phase, there seem to be more possibilities to implement specific measures to increase protein synthesis in order to achieve an increase in muscle mass and strength [40].

Other adjuvant strategies aimed at slowing down the catabolism of critically ill patients (beta-blockers, oxandrolone, anabolic steroids, promoting the enteral route, controlling intestinal dysbiosis, etc.) [38,41] have been considered, but none have demonstrated any clear benefits. On the other hand, proteomic characterization of skeletal muscle tissue as an endocrine organ directly involved in the regulation of metabolism has opened the door to a wide field of research.

### 3.2. Suitable Quantity and Quality of Protein-Energy Intake

**Protein quantity:** In a stressful situation, catabolic muscle loss can be avoided only if there is increased uptake of AA from the blood through intravenous infusion or digestion of enterally administered proteins, peptides or AA. These sources of AA can then stimulate protein synthesis to compensate for the accelerated rate of protein degradation and AA oxidation. In the literature, the consistent recommendation for protein intake is >0.8 g/kg/day, and the safe use of up to 2.5 g/kg/d is discussed [38,40,41,42]. Increasing protein doses beyond this threshold seems not to provide measurable benefits in muscle gain, but, based on studies in athletes, higher doses (4 g/kg/d) can be given without adverse effects [43]. It appears that the muscle synthesis process (MPS) is the main determinant of muscle hypertrophy, as it shows a saturable dose–response relationship with increased protein intake. We cannot give a single dose of protein to all patients, thus individualization on a patient-by-patient basis is critical. The main problem is that prescribed protein doses are not being administered, and we need to ensure adequate caloric intake (no overfeeding) so that protein utilization is correct [43]. 

**Protein quality:** Although no studies with nutritional intervention have been shown to be effective for improving any strength or function outcomes in critically ill patients at this time, the use of proteins with a high biological value appears to be fundamental for critically ill patients. Whey protein is the quintessential promoter of myofibrillar protein synthesis (MPS). This beneficial effect is attributed to the high proportion of leucine along with rapid digestibility and high bioavailability within plasma and muscle tissue. Whey protein is widely available, affordable and offers a relatively good safety profile.

Vitamin D, on the other hand, has varied functions in skeletal muscle, including calcium homeostasis, cell proliferation and differentiation, prevention of fat degeneration, protection against insulin resistance, and mobilization of arachidonic acid. The combination of whey protein and vitamin D may protect against sarcopenia and chronic inflammation in critically ill patients.

Essential amino acids (EAA) are the building blocks of muscle and play an important role in MPS. BCAA (branched-chain amino acids: leucine, isoleucine and valine) are considered fundamental, with leucine being the most important of these because it stimulates MPS via the mammalian target of the rapamycin (mTOR) signaling pathway and is also associated with the release of glyconeogenic precursors from the muscle. The dose of leucine to be administered in critically ill patients is unclear, but the benefit of EAA administration with high-dose leucine (5 g/day) has been demonstrated in diseases that cause muscle wasting, such as chronic diseases and atrophy from disuse. The PROT-AGE group’s recommendation for the anabolic threshold per meal of dietary protein/amino acid intake for the elderly population is 25 to 30 g of protein per meal, with approximately 2.5 to 2.8 g of leucine [44].

There are studies aimed at demonstrating the beneficial effect of a specific intervention such as hydroxymethylbutyrate (HMB) on muscle mass [45,46]. HMB is a product of leucine transamination produced in skeletal muscle that has been shown to improve MPS by its involvement in the mTOR pathway (similar to leucine), thereby decreasing the breakdown of muscle proteins independently of insulin. Under normal conditions, a portion of leucine is metabolized to HMB in the muscle cells; therefore, it would seem logical to think that HMB is more efficient in skeletal muscle renewal, but there is conflicting evidence regarding muscle strength and functional performance. HMB supplementation remains controversial because other studies, where a combined HMB/arginine/glutamine intervention on muscle loss was performed, do not demonstrate the same benefit [46].

Creatinine supplementation increases the availability of creatine and phosphocreatine in the muscle and supports anabolism by promoting the expression of growth factors, such as insulin growth factor (IGF)-1, and protein phosphorylation signaling.

### 3.3. Muscle Stimulation

Protein administration should be accompanied by resistance training for adequate muscle mass and strength recovery. Physical exercise should be considered a primary tool in the management of sarcopenia due to it having a significantly beneficial effect on anthropometric parameters and muscle function [38,39,42,47].

Exercise is defined as a “planned, structured, repetitive body movement intended to improve or maintain fitness”. Physical activity and exercise can be quantified in terms of FITT [42]: frequency, intensity, time (duration of the individual session and total duration of the program) and modality type (strength, endurance, etc.). In critically ill patients, resistance exercise is more complex due to the mitochondrial dysfunction and the reduced capacity for ATP regeneration/production, which is necessary for muscle contraction. 

Preparing customized training programs for each patient and starting to talk about exercise doses when in the ICU setting is starting to become critical to the clinical and functional outcome of the patient. Any barriers to exercise in the ICU should be removed, such as the design of ICUs not compatible with exercise or lack of physical therapists (or nursing professionals) to perform mobilization. Monitoring of the anabolism and mobilization program of critically ill patients is essential.

**f. Is it possible to monitor nutritional efficiency?**

As with any medical treatment, nutrition therapy aims to improve the clinical situation of critically ill patients. This is something that seems simple on the surface, but in practice it is not. 

Classically, laboratory parameters have been used to assess the nutritional status of critically ill patients, with the most commonly used parameters being total proteins, albumin, prealbumin, transferrin, retinol-linked protein, transthyretin, nitrogen balance and inflammatory parameters such as lymphocytes, C-reactive protein, interleukin-6 and tumor necrosis factor α [48]. None of them have been shown to be useful in assessing nutritional efficiency in critically ill patients. There are other far more complex measures that are currently not useful in routine clinical practice due to their inaccessibility and the time that the results take. Their use is limited to research [49]. 

In recent times, imaging techniques have been proposed for the assessment of nutritional performance, understood as the assessment of the amount of muscle mass, including bone densitometry, magnetic resonance imaging [50] and computed tomography [51]. Despite the potential usefulness of these techniques, in practice, the need to perform series, with the cost involved, and the need to mobilize patients, means that they are not often used. However, using ultrasound to measure muscle mass is the most widely used technique, as it is possible to perform it at the bedside, it is reproducible and all units have an ultrasound device. Its use requires knowledge of the protocol and its limitations of use [52]. The most examined muscle is the rectus femoris, for which both the quantity and quality of the muscle can be measured with specific software [53]. 

Another potentially useful technique is bioimpedance, which analyzes resistance and reactance to an alternating current being passed through the body. Hydration status and body composition can be assessed from these measurements. In critically ill patients, variations in acute hydration status limit its use [54]. 

In the absence of an accurate way to measure outcomes, monitoring of the nutritional process remains the best option. This monitoring includes:

- Reviewing the adequacy of the nutritional risk assessment.
- Evaluating the adequacy of the time of initiating nutrition therapy.
- Evaluating the adequacy of the prescription of nutrition therapy at both baseline and during treatment.
- Ensuring the adequate administration of prescribed doses or, otherwise, identifying situations where this administration can be optimized (e.g., assessing whether fasting is necessary before an imaging test).
- Ensuring monitoring of mechanical and metabolic complications associated with nutrition therapy and to provide solutions to them (including adequate glycemic control).
- Developing an early mobilization protocol and adhering to it to minimize the loss of lean mass.

On the other hand, the monitoring and optimization of nutrition therapy after discharge from the ICU must be performed by the professionals in charge at each center, which will allow for optimization of the nutritional process throughout the patient’s treatment. 

Therefore, given the current limitations on the monitoring of nutrition therapy, close monitoring of the nutritional process (each step of the process needed to perform nutrition therapy) is recommended.

**g. Key elements in transforming a care process**

Medical nutrition therapy is a care process that has an impact on patient prognosis [55,56,57]. If we want to include the SMARTFEEDING strategy as a care process that aims to increase the functional recovery of critically ill patients, we must consider three key aspects:

1. The purpose of the care process.
2. The key, unavoidable and applicable elements in the care process.
3. How to get the entire team to apply them, including team members who are not experts in medical nutrition therapy.

Applying the SMARTFEEDING strategy will have organizational implications on resource management at different levels of care, with clinical actions and attitudes beyond the patient’s location, and mainly associated with appropriate medical nutrition therapy and adjunctive physiotherapy. In addition, the multidisciplinary nature of this care process must be taken into account, with the intervention of the various professionals involved in clinical actions (physician, nurse, physiotherapist, dietitian, speech therapist, occupational therapist) and the different care settings. 

Although the ultimate objective is the functional recovery of critically ill patients, the transformation of the care process must take into account indicators of cost-effectiveness, which can be understood as clinical effectiveness. Consequently, for the objective of the change to be feasible, it must be appropriate to the resources, it must identify and address a need, and the results must be measurable. The key elements of the care process to be transformed are those that determine scientific evidence and consensus, which are highlighted in the care protocol as key indicators of the process [58,59]. 

To facilitate the implementation of complex protocols by all care teams, different strategies have been proposed. One of the most well-known and effective is the 4 Es (Engage, Educate, Execute, Evaluate) [60], which can be defined in terms of the following key points:

- Initiating a conceptual change in the care teams by circulating the care process to be transformed as well as the objective and impact that said transformation may have on the health of the patient.
- Educating non-experts on the essential processes of nutritional efficiency while understanding that many need to know a little (executors) and a few need to know a lot (leaders or reference persons).
- Conducting the protocol in different formats. It is useful to have three versions of the protocols available (extended, short and infographic), given that a simple format with few instructions and another broad and more explicit format where professionals can access more extensive information if needed is best suited to the different needs of participating healthcare providers. The bundle or package strategy of no more than five actions has been shown to be useful when applying some processes.
- Evaluating and defining which key indicators of the care process will be measured to monitor the process.
- Strong leadership, clearly identified in each unit in which it is implemented, along with resources and management recognition, is essential when carrying out transformation of integrated care processes. Leadership should not only design the strategy to facilitate implementation (motivation and education); it should be considered a visible resource that acts as the ultimate point of reference. Likewise, leadership must be responsible for analysis and evaluation, and may provide feedback to the care team that highlights the advantages of this transformation in the care process. These analysis actions should be considered as feeding into the motivation of the healthcare team itself. Training through clinical practice (e.g., daily checklists, weekly multidisciplinary sessions and periodic safety rounds) is intended to facilitate that purpose.

## 4. Concluding Remarks

Medical nutrition therapy is a care process that has an impact on patient prognosis. To ensure adequate nutritional management of critically ill patients, it is vital that the particular and changing nature of these types of patients is taken into account. Such uniqueness and variability require a careful and individualized approach aimed at promoting optimal functional recovery during and after the patient’s stay in the ICU. Our proposal is based on a comprehensive and dynamic strategy to enhance the functional recovery of the critically ill patient that considers both the nutritional status of the patient and modifiable circumstances. 

**Figure 2:**
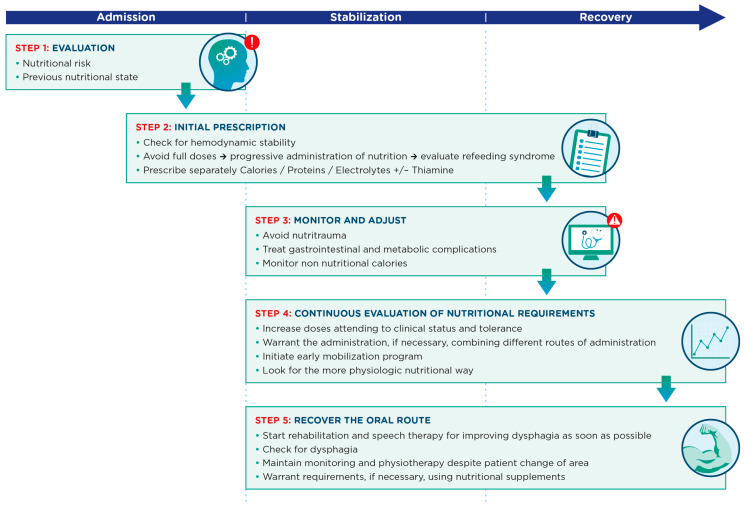
Key aspects in the nutritional management process for critically ill patients across admission, stabilization and recovery phases.

This strategy consists of several key aspects which are summarized in Figure 2. Firstly, medical nutrition therapy must be a strategic and targeted process requiring a medical history (prior weight loss, days without oral intake, physical activity, digestive system drugs, etc.) and an adequate baseline nutritional assessment that is recorded in the patient’s medical record and allows for timely initiation of medical nutrition therapy. In addition, this nutritional assessment should take into account (enabling identification and prevention of) associated risks, such as refeeding syndrome, gastrointestinal dysfunction or nutritrauma. A safe transition between the different access routes (enteral, parenteral and oral) for nutrition therapy should be ensured. Transfers between the ICU and other medical care units (care continuity) must ensure that information be included in the transfer in order to ensure optimal nutritional and functional recovery. 

One of the primary objectives of the resulting nutritional strategy will be to preserve lean mass in critically ill patients. Therefore, it is important to consider the different factors that affect and contribute to protein anabolism: metabolic characteristics of the phase the critically ill patient is in, adequate quantity and quality of protein intake, and adequate muscle stimulation. 

The correct implementation of such a strategy requires ongoing monitoring and support. However, we do not currently have any practical tools available that would allow us to adequately calculate nutritional efficiency in critically ill patients. This lack of an accurate measurement technique requires close and thorough monitoring of the nutritional process that covers all of the aforementioned aspects (with reassessment throughout the process).

Finally, medical nutrition therapy in critically ill patients is, by necessity, multidisciplinary and requires the dedication of an experienced team of intensivists, nutritionists, endocrinologists, nurses, physiotherapists, physiatrists, speech therapists, hospital pharmacists, etc. Following protocols is not enough; we need to focus on implementation, and the whole team needs to be properly trained and motivated. We believe that such a strategy has the potential to make a significant difference to the functional recovery of critically ill patients.

## Figures and Tables

**Table 1:** Recommendations and degree of consensus according to the ESPEN SOP.

Recommendations	Section	Degree of Consensus
1. The goal of nutrition therapy is to promote the maintenance of normal body function and the functional recovery of patients.	A. Objectives of clinical nutrition in critically ill patients	Strong consensus (98%)
2. The efficiency of nutrition therapy depends on patient-related factors, the management of injury, the quality of the nutritional prescription and the impact of contributing or harmful factors.	A. Objectives of clinical nutrition in critically ill patients	Strong consensus (100%)
3. The different evolutionary phases of critically ill patients create changes in their metabolic pattern, some of them without anabolism.	B. Critical patient phases	Strong consensus (98%)
4. The design of nutrition therapy is dynamic and should be reassessed at each phase.	B. Critical patient phases	Strong consensus (100%)
5. It would be useful to have a systematic assessment on hand that would help us identify which metabolic phase a critical patient is in.	B. Critical patient phases	Strong consensus (100%)
6. Life support techniques can alter nutritional efficiency in critically ill patients.	B. Critical patient phases	Strong consensus (92%)
7. The calculation of protein and caloric needs should be done on an individual basis and in consideration of co-morbidities, the patient’s usual weight, their BMI and the time of development (including physical therapy).	C. The quality of the nutritional process	Strong consensus (100%)
8. Within each center’s nutritional formulary, there must be a variety of formulas that are sufficient when it comes to meeting the specific needs of critically ill patients at each time point (hyperproteic, with or without fiber, hypercaloric, organ-specific, etc.).	C. The quality of the nutritional process	Consensus (87%)
9. Nutritional history should be included in the critically ill patient’s medical history: number of days without eating, weight loss, previous physical activity, use of “digestive” drugs, etc.	C. The quality of the nutritional process	Strong consensus (100%)
10. Caloric and protein intake calculation should take into account non-nutritional calories (e.g., propofol, citrate, serum) and increases in physiotherapy-related requirements.	C. The quality of the nutritional process	Strong consensus (97%)
11. Prescription does not guarantee that the patient’s requirements are met, so strategies should be implemented in order to ensure that the patient receives the total amount of the prescribed nutrition.	C. The quality of the nutritional process	Strong consensus (100%)
12. The enteral route is the route of choice in critically ill patients. Given the difficulty of achieving the prescribed requirements by the enteral route, and after optimizing tolerance, the parenteral route (complementary or total) should be considered.	C. The quality of the nutritional process	Strong consensus (92%)
13. In patients receiving an oral diet, monitoring of intake is recommended in order to identify patients in need of supplementation.	C. The quality of the nutritional process	Strong consensus (95%)
14. A diverse menu choice that increases patient satisfaction with food can help increase their intake and thus help improve nutritional status.	C. The quality of the nutritional process	Agreement in favor (72% in first round; 85.7% after review of the text)
15. Critically ill patients are at nutritional risk, which is aggravated if there is prior malnutrition.	D. Safety in prescribing medical nutrition therapy	Strong consensus (100%)
16. Inadequate administration of nutrients can lead to metabolic complications in critically ill patients (nutritrauma). Prevention is essential, especially in the early stages of nutrition therapy.	D. Safety in prescribing medical nutrition therapy	Strong consensus (100%)
17. Monitoring of physical, digestive or metabolic complications is essential, continuous and should be integrated into routine clinical practice.	D. Safety in prescribing medical nutrition therapy	Strong consensus (95%)
18. Tissue hypoperfusion markers should be included in the assessment at the beginning of enteral nutrition in critically ill patients.	D. Safety in prescribing medical nutrition therapy	Consensus (87%)
19. Medical nutrition therapy should not be initiated until critically ill patients are in a state of stabilized shock.	D. Safety in prescribing medical nutrition therapy	Strong consensus (97%)
20. The transition between nutrient access routes (PN, EN, ON) is a complex time that should follow protocol and be monitored in a specific manner.	D. Safety in prescribing medical nutrition therapy	Strong consensus (97%)
21. Before starting oral nutrition, the patient’s clinical condition must be adequate to test for dysphagia.	D. Safety in prescribing medical nutrition therapy	Strong consensus (21% in first round; 95% after review of the text)
22. Referral to other care services is a time of risk in which the continuity of medical nutrition therapy must be ensured.	D. Safety in prescribing medical nutrition therapy	Strong consensus (90%)
23. During the anabolic phase, protein contribution should be increased and combined with a rehabilitation program that promotes nutrient transformation to lean mass.	E. Determinants of food transformation into lean mass	Strong consensus (100%)
24. The use of high-quality proteins (digestibility, amino acid composition, etc.) favors nutritional efficiency.	E. Determinants of food transformation into lean mass	Favorable Agreement (66%)
25. It is critical to assess muscle dysfunction (MRC/MRC-SS, ICU mobility score, etc.) to categorize ICU patients based on their rehabilitation needs.	E. Determinants of food transformation into lean mass	Consensus (87%)
26. Critical patient rehabilitation must be a process integrated into their care, and it should follow protocol and be progressive and based on objectives.	E. Determinants of food transformation into lean mass	Strong consensus (97%)
27. Muscle and functional recovery of critically ill patients exceeds their ICU stay (and most likely their hospital stay), so continued medical nutrition therapy and rehabilitation outside the ICU is necessary.	E. Determinants of food transformation into lean mass	Strong consensus (100%)
28. Rehabilitation sessions range from passive treatment to the recovery of maximum function in the patient.	E. Determinants of food transformation into lean mass	Consensus (86%)
29. Physiotherapy sessions should increase in intensity in accordance with the patient’s tolerance at each time point. Rest is an essential part of the rehabilitation strategy.	E. Determinants of food transformation into lean mass	Consensus (58% in first round; 76.2% after review of the text)
30. Devices that facilitate recovery of lean mass, e.g., cycloergometers, standing frames, walking slings, etc., must be incorporated.	E. Determinants of food transformation into lean mass	Agreement in favor (69%)
31. Currently, we do not have tools that would allow us to adequately calculate nutritional efficiency in critically ill patients.	F. Is it possible to monitor nutritional efficiency?	Strong consensus (94%)
32. In the absence of a key indicator of nutritional efficiency, close monitoring of the nutritional process should be performed to optimize its results.	F. Is it possible to monitor nutritional efficiency?	Strong consensus (94%)
33. Imaging techniques could play a very important future role in the monitoring of muscle quantity and functional quality.	F. Is it possible to monitor nutritional efficiency?	Strong consensus (94%)
34. Medical nutrition therapy is a care process that has an impact on patient prognosis.	G. Key elements in transforming a care process	Strong consensus (91%)
35. Medical nutrition therapy in critically ill patients is, by necessity, multidisciplinary and requires the dedication of intensivists, nutritionists, endocrinologists, nurses, physiotherapists, physiatrists, speech therapists, hospital pharmacists, etc., integrated into the team and with experience in managing this type of patient.	G. Key elements in transforming a care process	Strong consensus (95.2%)
36. It is necessary that we go beyond following protocols. To this end, the following aspects are critical: 1. The formation and motivation of the multidisciplinary team involved in the process. 2. Having a simplified version of the protocol and a definition of process indicators on hand.	G. Key elements in transforming a care process	Strong consensus (95.2%)
37. It is necessary to identify leaders who promote training, evaluation, analysis and feedback as essential parts of the process.	G. Key elements in transforming a care process	Strong consensus (100%)
38. Each unit must have a reference person that coordinates this process. This figure must be recognized by managers.	G. Key elements in transforming a care process	Consensus (81%)
39. Adapting protocols to different formats or models of care aids their implementation.	G. Key elements in transforming a care process	Consensus (82%)
40. Training through clinical practice is a useful tool that benefits from different strategies: daily checklists, weekly multidisciplinary sessions and periodic safety rounds	G. Key elements in transforming a care process	Strong consensus (100%)

**Table 2:** Definition of Disease Phases in the Course of Critical Illness.

Disease Phase		Organ Dysfunction	Inflammation	Metabolic State	Approximate Duration/Period (Days)
Acute phase	Early acute phase	Severe or increasing (multiple) organ dysfunction	Progressive inflammation	Catabolic	1–3
	Late acute phase	Stable or improving organ dysfunction	Regressive inflammation	Catabolic-anabolic	2–4
Post-acute phase	Convalescence/rehabilitation	Largely restored organ function	Resolution of inflammation	Anabolic	>7
	Chronic phase	Persistent organ dysfunction	Persistent immune suppression	Catabolic	>7

## Data Availability

All the data supporting the findings of this study are fully included within this published article. There are no additional datasets.
